# Natural Experience Modulates the Processing of Older Adult Faces in Young Adults and 3-Year-Old Children

**DOI:** 10.1371/journal.pone.0057499

**Published:** 2013-02-27

**Authors:** Valentina Proietti, Antonella Pisacane, Viola Macchi Cassia

**Affiliations:** Department of Psychology, Università degli Studi di Milano-Bicocca, Milano, Italy; University of Leuven, Belgium

## Abstract

Just like other face dimensions, age influences the way faces are processed by adults as well as by children. However, it remains unclear under what conditions exactly such influence occurs at both ages, in that there is some mixed evidence concerning the presence of a systematic processing advantage for peer faces (own-age bias) across the lifespan. Inconsistency in the results may stem from the fact that the individual’s face representation adapts to represent the most predominant age traits of the faces present in the environment, which is reflective of the individual’s specific living conditions and social experience. In the current study we investigated the processing of younger and older adult faces in two groups of adults (Experiment 1) and two groups of 3-year-old children (Experiment 2) who accumulated different amounts of experience with elderly people. Contact with elderly adults influenced the extent to which both adult and child participants showed greater discrimination abilities and stronger sensitivity to configural/featural cues in younger versus older adult faces, as measured by the size of the inversion effect. In children, the size of the inversion effect for older adult faces was also significantly correlated with the amount of contact with elderly people. These results show that, in both adults and children, visual experience with older adult faces can tune perceptual processing strategies to the point of abolishing the discrimination disadvantage that participants typically manifest for those faces in comparison to younger adult faces.

## Introduction

Adults are “experts” at processing faces. It is well established, however, that this expertise exhibits strong biases, such that adults’ processing abilities are greater for some categories of faces than for others, with relevant categories including species (e.g. [1]), race (e.g. [2]), gender [3], and age (see [4] for a review). The present study focuses on variations in face processing abilities as a function of face age. Age is one of several sources of information that is rapidly extracted from faces by adults as well as by children [5] and is one important dimension that influences how faces are attended to (e,g., [6]), encoded (e.g., [7], [8]) and retrieved from memory (e.g., [9], [10]). However, it remains unclear under what conditions exactly such influence occurs.

In several different kinds of face-processing tasks, ranging from eyewitness identification to perceptual discrimination, adults exhibit better performance for faces within their own age group compared to both other adult faces outside the observer’s age range [9], [11]–[15] and infant [16], [17], [18] or child faces [16], [19], [20], [21]. This evidence (see [4] for a meta-analytic review) has led researchers to propose the existence of an own-age bias (OAB, also called other-age effect; [10], [22]) in face processing, which would parallel other in-group biases such as that for faces of our own species (e.g., own-species bias; [23]) or race (i.e., own-race bias; see [24] for a review).

Importantly, because age, unlike species and race, is a changeable dimension inherent to faces, as well as to the beholder’s status, one crucial prediction arising from the hypothesis of an OAB in face processing would be that of a consistent shift in processing bias for faces of increasing age across the lifespan. Nevertheless, direct evidence of the existence of a systematic advantage in the processing of peer faces in populations other than young adults is sparse. A number of studies have failed to observe an OAB in elderly participants (e.g., [12], [13], [15]), and research with children has yielded a very mixed set of results. Specifically, children older than 5 years were found to manifest a recognition memory advantage for peer faces in at least four studies employing an intentional old/new recognition memory task [19], [25], [26], [27], whereas they failed to show an OAB in six other studies using different types of tasks (i.e., implicit memory, [25]; old/new recognition memory, [28], [29], [30]); verbal person memory, [31]; two-alternative forced choice recognition memory [32]). These inconsistencies in the results of available research suggest that, if present, the OAB observed in adults is much less reliable within other age ranges.

Interpretations of the OAB mimic those posed to account for other in-group biases, such as the own-race bias, and can be grouped in two broad categories: one focusing on perceptual learning processes, and the other focusing on social cognitive mechanisms. Both accounts share the idea that intergroup contact has some influence on the magnitude of face processing biases, whereas they differ for the specific cognitive mechanisms through which contact might actuate this influence (see [24] for a review). The perceptual learning account suggests that extensive exposure to a given face category (e.g., human faces, own-race faces) leads to a well-refined prototype and to enhanced sensitivity to the featural and configural cues that differentiate individual faces from this category (e.g., [8], [33]). The social cognitive account, on the other hand, posits that the processing strategies we use to encode a given face variy as a function of the in-group or out-group status of that face, with intergroup contact and experience setting the criteria for in-group and out-group membership. While in-group faces are processed at the individual level, out-group faces are processed at the more superficial category level, by focusing on category-specifying features at the expenses of individuating information or through reduced motivation to attend to relevant individuating features [34], [35], [36], [37].

With regards to the own-race bias, recent studies suggested that perceptual expertise, social categorization processes and motivational factors work together to promote the recognition advantage for own-race compared to other-race faces (e.g., [38], [39]). Instead, the contribution of these factors to age-related biases is still debated and direct evidence suggesting the influence of in-group/out-group categorization on adults’ recognition of own-age and other-age faces is sparse (e.g., [7]). Notably, the main issue within this debate is whether age truly acts as an own-group factor in the same way as race does.

In fact, the labeling of the age bias exhibited by adults as “own” reflects the assumption that, as for species and race, individuals are mostly exposed to faces within their own age group. However, this assumption underestimates individual differences in the amount of differential passive and active experience with individuals of various ages that naturally occurs in people’s life. Indeed, it has been proposed that discrepancies in age biases manifested by elderly adults may depend upon differences in living conditions (retirement communities vs independent housing), which may differentially constrain contact with people of various age groups (e.g. [11], [15]). In accord with this hypothesis, there is some evidence that, in both younger and older adults, the amount of self-reported social exposure to own- and other-age individuals is related to the size of the OAB [7], [40]. In addition, adults who accumulated extensive experience with newborns (i.e., maternity-ward nurses) or children (i.e., school teachers) showed enhanced discrimination/recognition and processing skills for infant [18] and child faces [8], [16], [20], [21] compared to non-experienced age-matched controls (see [41] for a review).

In a similar vein, it has been recently reported that young children’s processing skills for infant and child faces vary as a function of whether or not children have a younger or an older sibling in their home [17], [42]. In these studies, 3-year-old children without a younger or an older sibling were more accurate at discriminating adult faces compared to infant or child faces. In contrast, children with a younger sibling were equally skilled at differentiating adult and infant faces [17] and, similarly, those with an older sibling, unlike first-born children, showed comparable discrimination abilities for adult and child faces [42]. These findings were interpreted as suggesting that adult faces are over-represented in young children’s face space (see also [43]), unless extensive experience with individuals from other age groups occurred.

This evidence seems to suggest that, in fact, there might not be such a thing as an OAB in face processing, which remains stable across all developmental age ranges. Rather, age biases across the lifespan could be conceived as arising from the constant adaptation of face representation to reflect the predominant age traits of the faces that are present in the individual’s social environment, which often, but not always, happen to be peer faces (see [44]). In case of young adults social experience is heavily biased towards same-aged individuals, unless living conditions and/or working experiences induce frequent contact with other age groups. In case of young children perceptual experience is more likely biased towards caregivers and other adult individuals than towards peers, although under particular conditions it may extend as well to individuals of other ages.

Our purpose in the current study was to provide further evidence for the influence of social and perceptual experience on adults’ and children’s face processing skills to another category of adult individuals, namely elderly adults. To this end, we compared perceptual processing strategies and discrimination abilities for younger and older adult faces in two groups of young adults (Experiment 1) and two groups of 3-year-old children (Experiment 2) differing in the amount of experience accumulated with elderly adults. To place emphasis on the encoding stage of visual processing and limit memory demands for the participants, we tested both adults and children in a delayed two-alternative forced choice matching-to-sample task, in which they were asked to match a briefly presented target face to two simultaneously presented test faces appearing after a short delay. Moreover, to investigate the effects of experience on the perceptual processing strategies adopted by participants in discriminating younger and older adult faces, we measured the size of the inversion effect [45] for the two types of faces.

There is a general consensus that specific processing strategies are involved in face recognition, which are linked to the acquisition of perceptual expertise [46]. In fact, although both configural and featural information contributes to facial recognition, it is well established that configural processing occurs more for faces and other stimuli of particular expertise [47]. Configural processing involves the extraction of the relations among the stimulus features. As for faces, it includes the detection of first-order relations that specify the basic geometry that all faces or face-like stimuli share, holistic processing that integrates facial features into a gestalt, and sensitivity to second-order relations that specifies differences among individuals in the spacing of internal features (see [48]). Detection of configural information drops abruptively when faces are inverted, giving rise to the well-known face inversion effect [45], which is commonly used as a measure of perceptual expertise. Accordingly, the stronger or selective inversion effect observed for own-race versus other-race faces (e.g., [49]) and for own-age versus other-age faces (e.g., [16]) in adults has been interpreted as arising from asymmetrical race and age experience. The inversion effect has been reported to be face-specific in children as young as 3 years [50]. At this age, like in adulthood, the effect is modulated by asymmetrical race and age experience, since it is larger for own-race than other-race faces [51] and for adult faces than newborn or child faces, unless experience with a younger or older sibling occurred [17], [42]. Based on this earlier evidence, we used the size of the inversion effect in the current study as a measure of perceptual learning engendered by experience with elderly adult individuals.

For the purpose of the current study, “elderly adults” were defined as being older than 60 years of age. Faces of elderly adults were chosen because, although the amount of each participant’s active experience with individuals from this specific age group can still be quantified, sporadic, non-intentional contact with elderly people is common in our everyday environment, thus it is likely that passive exposure to older adult faces occurs for virtually any child or adult individual. Moreover, although older adult faces differ from younger adult faces on a number of features (e.g., [52]), they also share more relevant perceptual characteristics with younger faces than infant and child faces do [53]. Therefore, the finding of better discrimination of younger compared to older adult faces in adults with limited exposure to elderly people in Experiment 1 would provide further evidence for the robustness of the OAB in adults. At the same time, though, the investigation of whether the OAB is abolished or at least mitigated in adults who recently accumulated extensive experience with elderly adults would add to existing demonstrations that face representation retains enough flexibility into adulthood so as to adapt to newly encountered face-age groups.

In Experiment 2, the finding of a perceptual processing advantage for younger adult faces in 3-year-old children when these faces are compared to another sub-class of adult faces would add to extant demonstrations that younger adult faces are over-represented in young children’s face space, possibly resulting from early and repeated experience acquired with adult caregivers. Nevertheless, it is also possible that young children perceive younger and older adults as part of the same broad social group, thus generalizing their motivation to actively seek for experience with faces of adult caregivers to elderly people. If this were the case, we would expect that, even in the absence of extensive exposure to elderly people, 3-year-old children were equally good at discriminating younger and older adult faces. In any case, like for adults, the comparison between children with different amounts of contact with elderly individuals would provide further demonstration that the developmental trajectory of age biases in face processing are directly related to the amount of differential environmental exposure that children have or have had with faces of different ages.

## Experiment 1

### Method

#### Participants

The sample included 36 adult females, 18 in the *low-experienced* control group (mean age = 34 years; range = 23–53 years) and 18 in the *high-experienced* group (mean age = 44.8 years; range = 30–57 years). All participants were screened prior to testing via a questionnaire, which included specific enquiries aimed at assessing if, in the past year, they have been living with parents and/or grandparents older than 60 years, if they have had contact with parents or grandparents of friends or acquaintances, and if they have had a job (full-time or otherwise), which put them in contact with elderly people. Participants were included in the low-experienced group if, in the last year, they had not acquired more than 500 hours of experience (see [16] for a similar selection criterion applied to adult novices of newborns and children) (see Table 1). Participants in the high-experienced group were nursing home assistants working full time or part time (working hours per week: M = 34; range = 21–55) in a retirement home. All had a working experience of at least 5 years (M = 14 years range = 5–25), and had acquired more than 1000 hours of experience with elderly people within the past year (see Table 1).

**Table 1:** Questionnaire-based information concerning the amount of contact that participants in the low-experienced and in the high-experienced group had with elderly adults aged 60 to 90 years.

Low-Experienced Group		High-Experienced Group		
Subject no.	Tot. hr contact in last year	Subject no.	Tot. hr contact in last year	Tot. hr working experience
1	96	1	2640	20
2	24	2	1248	13
3	96	3	2736	12
4	12	4	1968	11
5	0	5	3408	19
6	192	6	2400	12
7	72	7	1824	14
8	288	8	1872	9
9	108	9	2400	24
10	20	10	2328	12
11	96	11	1680	10
12	288	12	1200	13
13	0	13	3456	25
14	336	14	1920	5
15	60	15	2544	12
16	288	16	3888	14
17	48	17	1296	24

For each participant, the table reports the total amount of hours of contact in the last year. For the nursing home assistants in the high-experienced group, the number of years of working experience is also reported.

#### Ethics statement

All procedures used in the current study complied with the Ethics Standards outlined by the University of Milano-Bicocca. Informed written consent was obtained before testing from all participants involved in the study.

#### Stimuli

**Figure 1:**
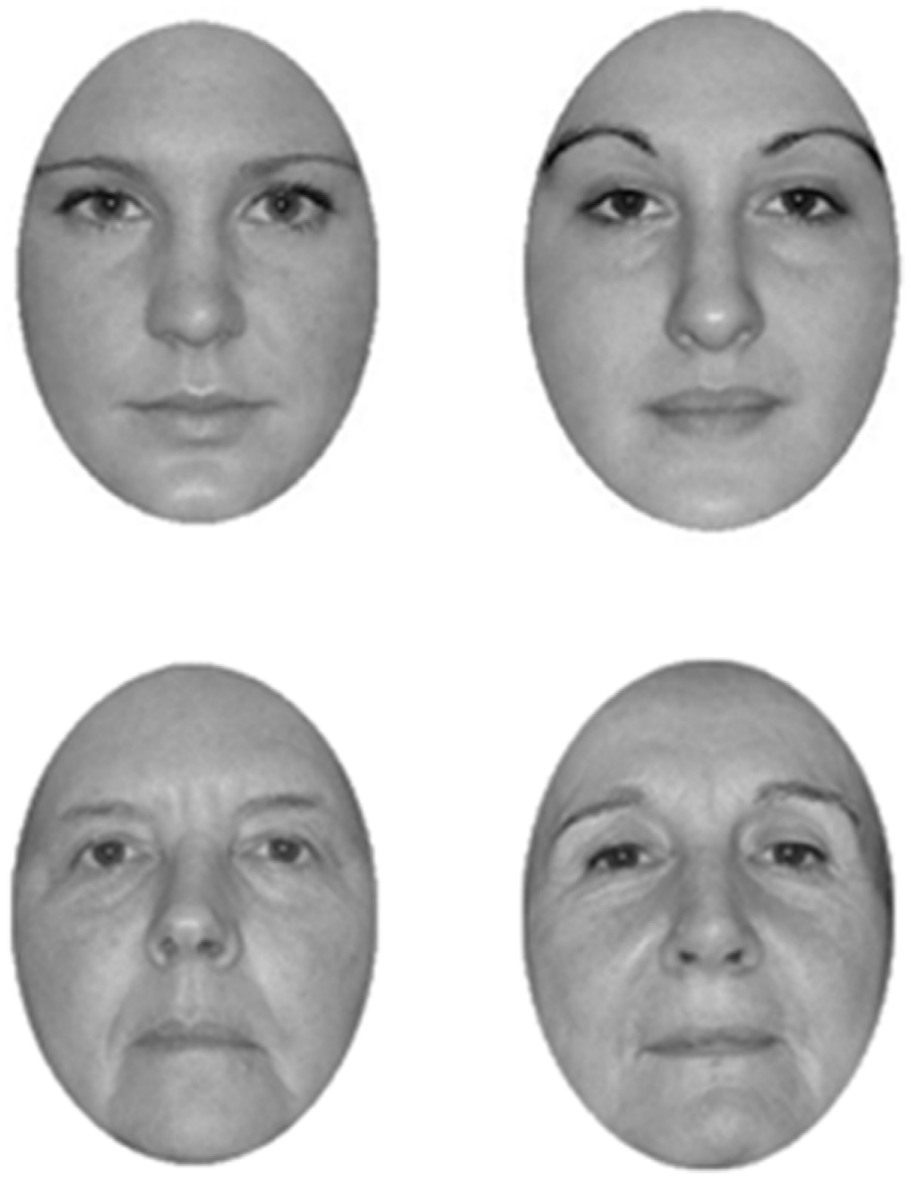
Examples of the younger adult and older adult faces stimuli used in the study.

Stimuli consisted of 48 gray-scale photographs of younger adult (20–30-year-old) faces and 48 older adult (60–90 year-old) faces that displayed full-front neutral expressions and were unfamiliar to the participants (Figure 1). To control for possible interfering effects of gender, all faces were female, so that stimulus gender was matched to participants’ gender. Older faces were taken from Minear and Park [54], whereas younger adult faces were taken from our own database [16]. Face images were cropped in a standard oval, eliminating cues from external features such hair, ears and neck. All faces subtended a horizontal visual angle of 4.43° and a vertical angle of 6.04° when viewed from approximately 40 cm and appeared on a grey background. An attempt was made to pair faces based on subjective criteria of luminance and overall similarity, so as to generate 24 pairs for each face age. An additional 16 face images (8 for each face age) were used as stimuli in the practice trials. Inverted stimuli were created by a 180° rotation of each face.

#### Procedure

Participants were tested individually on a portable computer using a delayed two-alternative forced-choice matching-to-sample task. They were told that one face, the target, would appear on the screen and that they would be asked to recognize that target between two faces, the probes, appearing after the initial presentation. The target face was presented centrally for 1 s, followed by a 500-ms blank inter-stimulus interval (ISI) and then the two probe stimuli, the target face and a novel face, that appeared side by side. The participants’ task was to respond as quickly and accurately as possible by pressing a key on the keyboard corresponding to the side of the screen on which the target face appeared. The two probe stimuli remained on the screen until a response was made; after that an inter-trial interval of 500 ms elapsed before the start of a new trial. The left or right position of the target and novel faces was counterbalanced across trials. The target face and the two choices appeared in same orientation.

The experiment consisted of eight blocks of trials, two for each face age (younger adult, older adult) and orientation (upright, inverted) condition. There were 24 trials per block, for a total of 48 trials per condition and an overall total of 192 trials. Upright and inverted trials were administered in two sessions separated by a 15-min break. All participants were tested with upright trials first, whereas face age was alternated between blocks, with the age of the faces in the first block counterbalanced across subjects. At the beginning of each session, we gave participants 8 practice trials (4 for each face age condition) to ensure that they understood the task. Responses on practice trials were not considered. Response accuracy and response times (RTs) to correct responses on test trials were recorded as dependent variables.

### Results

To compare recognition performance of participants in the two groups we analyzed accuracy rates and response times to correct responses separately in two 2×2×2 repeated-measures Analyses of Variance (ANOVAs) with face age (younger adult, older adult) and orientation (upright, inverted) as within-participants factors, and experience group (low-experienced, high-experienced) as between-participants factor.

#### Accuracy rates

**Figure 2:**
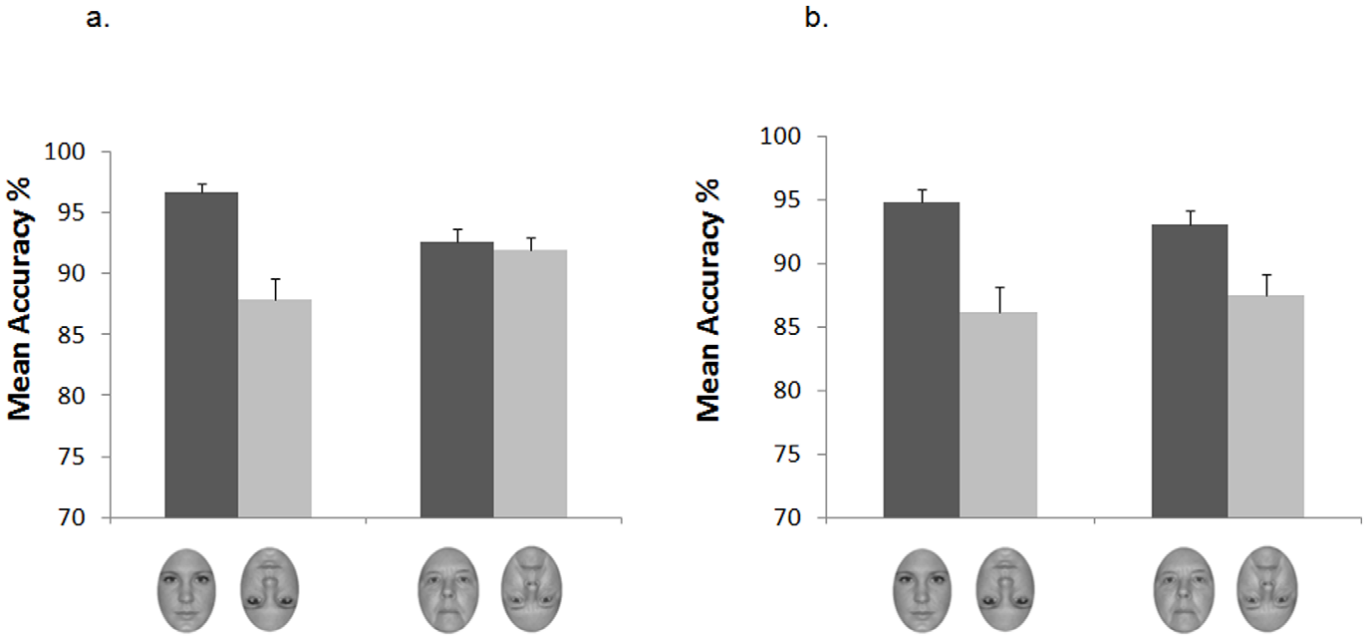
Mean accuracy rates of adult participants in the low-experienced group (a) and in the high-experienced group (b) when younger and older adult faces were presented upright and inverted. Error bars represent standard errors of the means.

The analysis of mean accuracy rates revealed a significant main effect of orientation, *F*(1,34) = 52.13, *p*<.001, *η^2^* = .605. This main effect was qualified by a significant Face Age × Orientation interaction, *F*(1,34) = 21.47, *p*<.001, *η^2^* = .387, as well as by a significant three-way interaction between face age, orientation and experience group, *F*(1,34) = 4.18, *p*<.05, *η^2^* = .109, indicating that accuracy for younger adult and older adult faces in the two orientation conditions differed for the low-experienced and the high-experienced group. To further explore this interaction, separate 2×2 ANOVAs were performed on accuracy data for each group of participants. While a main effect of orientation was found for both the low-experienced group, *F*(1,17) = 23.95, *p*<.001, *η^2^* = .585, and the high-experienced group, *F*(1,17) = 28.82, *p*<.001, *η^2^* = .629, the critical Face Age × Orientation interaction was present only for the low-experienced group, *F*(1,17) = 19.77 *p<.001*, *η^2^* = .538. Planned comparison revealed that these participants were better at discriminating upright younger adult faces (M = 96.7%) compared to upright older adult faces (*M = *92.6%), *t*(17) = 5.66, *p<*.001, and showed an opposite discrimination advantage for older adult faces over younger adult faces in the inverted orientation (*M = *92% vs 87.9.%), *t*(17) = 2.25, *p*<.05. Accordingly, they showed a significant inversion effect for younger adult faces (*M = *96.7% vs 87.9%), *t*(17) = 5.14, *p*<.001, but not for older adult faces (*M = *92.6% vs 92.0%) (*p*>.4) (Figure 2a). In contrast, nursing home assistants in the high-experienced group showed a significant decrement in performance on inverted compared to upright trials for both younger adult faces (upright: *M = *94.9% vs. inverted: *M* = 86.2%), *t*(17) = 5.33, *p*<.001, and older adult faces (upright: *M = *93.1% vs. inverted: *M* = 87.6%), *t*(17) = 3.76, *p*<.005, resulting in an inversion cost (computed by subtracting the mean accuracy for the inverted condition from the mean accuracy for the upright condition) of similar magnitude for the two face sets (younger adult faces: *M = *8.7%, older adult faces: *M = *5.5%). Nurses’ discrimination performance did not differ for younger adult and older adult faces in either the upright (*M = *94.9% vs 93.1%) (*p*>.09) or the inverted condition (*M = *86.2% vs 87.6%) (*p*>.42) (Figure 2b).

To complete the exploration of the differences between the two groups, we directly compared accuracy rates for each condition and found the comparison to be significant only for the inverted older adult face condition, *t*(34) = 2.32, *p*<.005 (all other *p*s >16.). Finally, between-group comparisons of the size of the inversion cost for each face age revealed that, for older adult faces, the inversion cost was significantly larger in the high-experienced group than in the low-experience group (*M = *5.5% vs 0.6%), t(34) = 2.95, *p*<.01, whereas for younger adult faces the effect was not significantly different in the two groups (*p*>.96).

#### Response times

**Figure 3:**
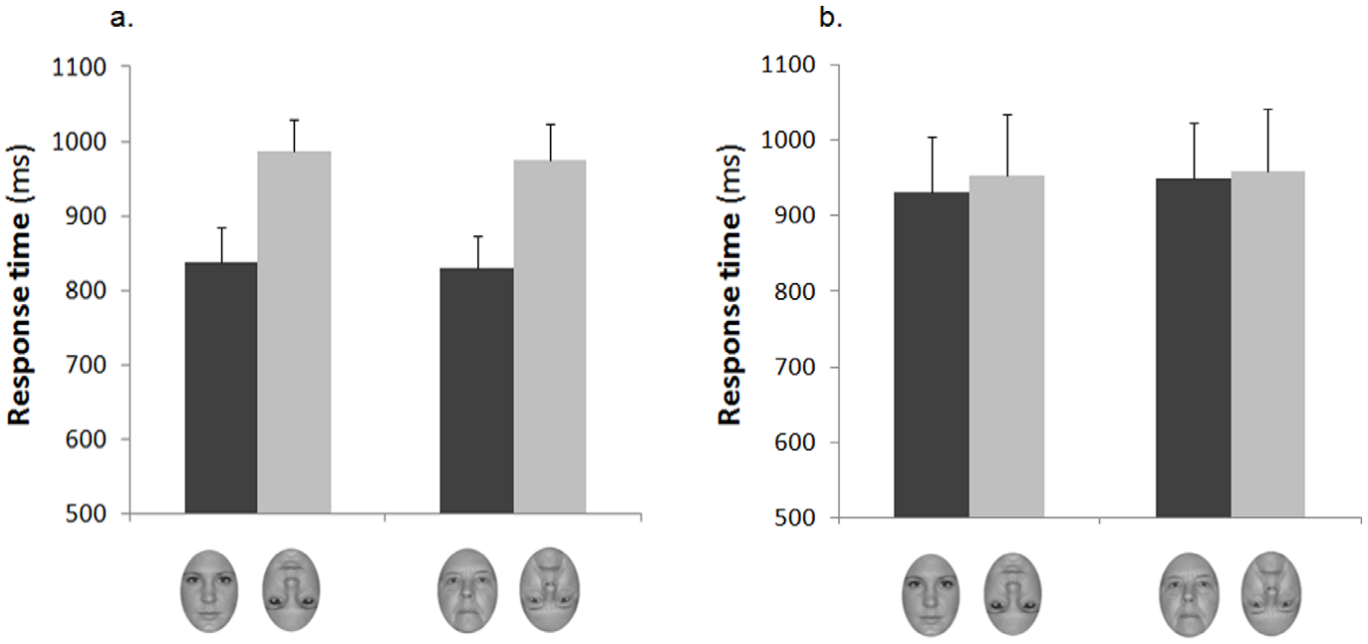
Mean response times to correct responses for the low-experienced group (a) and the high-experienced group (b) for upright and inverted younger and older adult faces. Error bars represent standard errors of the means.

The 3-way ANOVA on correct response times revealed a significant main effect of Orientation, *F*(1,34) = 14.67, *p*<.001, *η^2^* = .301, which was qualified by a significant two-way interaction between orientation and experience group, *F*(1,34) = 9.64, *p<*.005, *η^2^* = .221. This interaction was further explored through separate 2×2 ANOVAs on RT data from each group. The ANOVA performed on the low-experienced group revealed a significant main effect of orientation, F(1,17) = 15.39, *p*<. 001, *η^2^* = .475, due to overall faster responses to upright (*M* = 887.2 ms) compared with inverted (*M* = 968.3 ms) faces, whereas the interaction between face age and orientation was not significant (*p*>. 87 (Figure 3a). The ANOVA performed on the high-experienced group did not reveal any significant main effect or interaction (all *p*s >.25) (Figure 3b).

### Discussion

Results showed that a quantifiable amount of contact with elderly individuals in adulthood can modulate the ability to discriminate individual faces from this age group as well as the perceptual processes used to perform such discrimination.

Accuracy data showed that adults who had limited contact with elderly individuals were better at discriminating younger adult faces compared to older adult faces in the canonical upright condition, and exhibited an inversion effect which was specific for younger adult faces. Unlike low-experienced participants, nursing home assistants were equally proficient at discriminating upright younger and older adult faces, and manifested an inversion effect of equal magnitude for both face ages. Although analyses of response times proved that this pattern of results could not be explained by a speed-accuracy trade-off, response time data were less sensitive in discriminating the effects of face age and experience across the two groups. In fact, correct responses provided by low-experienced participants were as fast to upright younger adult faces as they were to upright older adult faces and were slower on inverted compared to upright trials for both face types. Moreover, the time it took to the nurses to provide their correct responses did not differ across conditions.

Overall, the finding that, in the low-experienced participants, stimulus inversion consistently affected both discrimination accuracy and response times to younger adult faces whereas it only affected response times to older adult faces indicates that the inversion effect in these participants was more robust for the former than for the latter type of faces. This suggests that low-experienced participants were better able to extract the relevant featural/configural cues necessary for efficient face recognition from younger adult faces, whereas they possibly relied more heavily on featural cues, such as wrinkles and eye shape, to efficiently discriminate older adult faces. Most crucially, the finding of a generalized inversion effect for younger and older adult faces in the high-experienced participants indicates that perceptual experience improved nursing home assistants’ sensitivity to the featural/configural cues embedded in older adult faces, mirroring the sensitivity that they have for these same cues in younger adult faces. The demonstration that, unlike low-experienced participants, the nursing home assistants did not show an own-age discrimination bias, being equally good at discriminating upright younger and older adult faces, lends further support to this conclusion.

Of note, between-groups comparisons showed that, although nursing home assistants were significantly less accurate than low-experienced adults at discriminating inverted older adult faces, they did not show a corresponding improvement in the discrimination of upright older adult faces. Although not predicted, this finding was not completely unexpected, since it is not new in the literature. The finding that experience acquired in adulthood with a specific stimulus category can induce the emergence of an inversion effect without producing a significant increase in discrimination or recognition performance on upright trials has been reported previously in the seminal study conducted by Diamond and Carey ([54], Exp. 2 and 3) with dog experts, as well as in a more recent study investigating the effects of experience on adults’ recognition of newborn faces [18]. In this recent study, maternity-ward nurses, unlike novices, manifested an inversion effect of the same magnitude for adult and newborn faces, without being able to perform equally well at discriminating the two face categories in the upright orientation. In Diamond and Carey’s [55] study, dog experts were significantly worse at recognizing inverted dog images with respect to the novices, while showing with no corresponding increase in recognition accuracy for upright dog trials. Our results add to this evidence in suggesting that experience acquired in adulthood can tune visual processes involved in face recognition for use with newly experienced classes of faces, but that is not sufficient to produce a corresponding improvement in discrimination/recognition abilities for those faces.

Overall, results of Experiment 1 allowed us to confirm that, in the absence of extensive experience, young adults exhibit a perceptual processing advantage for own-age faces over faces of older adults, and that extensive exposure to elderly adults can eliminate this advantage. In Experiment 2, we aimed to extend these findings to 3-year-old children, to investigate whether younger adult faces are processed more efficiently than older adult faces even when they do not match the age of the beholder, and whether experience with elderly people can modulate the age bias in children in the same way as in adults. To this end, we tested two groups of 3-year-old children with different amounts of experience with elderly people for their recognition of upright and inverted younger adult and older adult faces.

## Experiment 2

### Method

#### Participants

The final sample consisted of 36 3-year-old children (20 females; mean age = 3 years 7 months, range = 3 years 1 month – 3 years 11 months), 18 in the low-experienced group, and 18 in the high-experienced group. Three additional children were tested, but excluded from the sample due to failure to reach criteria established for data analyses (see Results). Children’s assignment to each group was based on parents’ responses to a questionnaire that included specific enquiries aimed at assessing the amount of contact that children have or have had with grandparents and other people older than 60 years from the time of their birth. On the basis of parents’ responses, we calculated the total amount of hours each child had contact with elderly people within each year over the past three years. Children were included in the low-experienced group if they had not acquired an average of more than 500 hours of experience per year over the past three years (see Table 2). Children assigned to the high-experienced group had acquired an average of more than 1000 hours of experience with elderly people within the same time frame (see Table 2).

**Table 2:** Questionnaire-based information concerning the amount of contact that 3-year-old children in the low-experienced and in the high-experienced group had with elderly adults aged 60 to 90 years. For each participant, the table reports the average number of hours of contact per year during the last 3 years.

Low-Experienced Group		High-Experienced Group	
Subject no.	Average hr contact	Subject no.	Average hr contact
1	528	1	1164
2	144	2	2256
3	12	3	1725
4	432	4	2125
5	368	5	2480
6	156	6	1600
7	344	7	1640
8	480	8	1955
9	306	9	2225
10	300	10	1015
11	410	11	1584
12	369	12	1356
13	70	13	1587
14	280	14	1867
15	453	15	1144
16	144	16	2712
17	459	17	1782
18	528	18	1423

#### Ethics statement

All procedures used in the current study complied with the Ethics Standards outlined by the University of Milano-Bicocca. All participants’ parents gave informed written consent prior to commencement of the study, and children gave their verbal assent before testing.

#### Stimuli and procedure

Stimuli were the same as in Experiment 1, with the exception that only 48 (12 pairs for each face age) of the original 96 face images were used. Unlike Experiment 1, no efforts were made to keep the relation between stimulus gender and participants’ gender constant, as all faces were female and both boys and girls were included in the sample. This was done in light of studies on gender development indicating that complete gender constancy is achieved not earlier than 6 years of age [56]. This makes it unlikely that, in 3-year-old children, gender acts as a grouping factor driving social categorization processes that have been found to affect face encoding (e.g., [39]). The procedure was also the same as in Experiment 1 except as follows. Exposure time of the target face was extended to 5 s, the number of blocks and trials per condition were reduced to 4 and 12, respectively, for an overall total of 48 trials, the length of the break between the two testing sessions ranged from 1 to 24 hours, and children were invited to provide their response either by pointing to the target or pressing a computer key. The experimenter determined the start of the next trial by pressing the mouse, and response accuracy was recorded as the dependent variable.

### Results

Data were included in the analyses only if the child was correct on more than 50% of upright younger adult trials; 3 children were excluded because they did not meet this criterion. For the remaining children in each group, one-sample *t*-tests confirmed that accuracy was significantly above chance (i.e., 50%) for both face ages in the upright orientation condition (all *p*s<.005).

**Figure 4:**
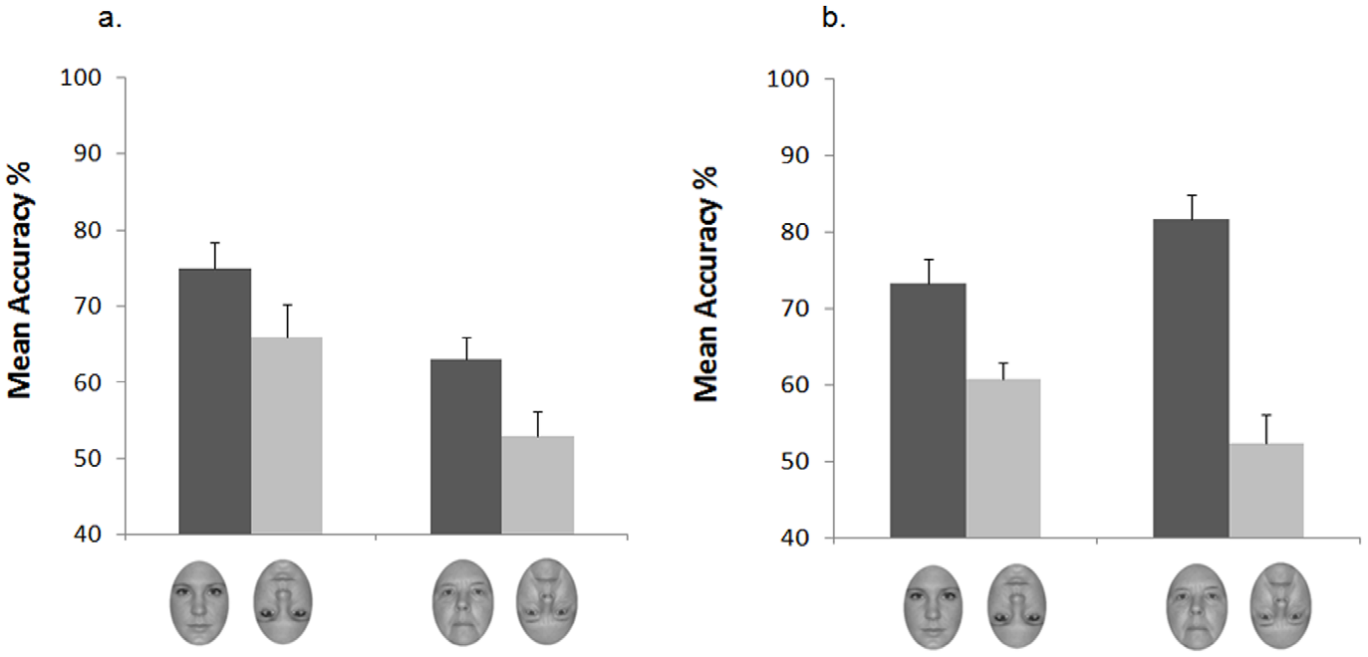
Mean accuracy rates of 3-years-old children in the low-experienced group (a) and in the high-experienced group (b) when younger and older adult faces were presented upright and inverted. Error bars represent standard errors of the means.

To compare discrimination performance of children in the two groups we performed a preliminary ANOVA with face age (younger adult, older adult) and orientation (upright, inverted) as within-participants factors, experience group (low-experienced, high-experienced) as the between-participants factor and gender (male, female) as an additional factor. The ANOVA revealed no main effect or interactions involving gender, and data were consequently collapsed across this factor in the subsequent three-way ANOVA. The analysis revealed significant main effects of face age, *F*(1,34) = 6.36, *p*<.05, *η^2^* = .158, and orientation, *F*(1,34) = 46.71, *p*<.001, *η^2^* = .579, which were both qualified by a marginally significant Face Age × Orientation interaction, *F*(1,34) = 4.07, *p = *.051, *η^2^* = .107. The interaction between face age and experience group was also significant, *F*(1,34) = 6.20, *p*<.05, *η^2^* = .154, indicating that discrimination accuracy for younger and older adult faces differed for the two groups of children. To further explore this interaction, we performed separate 2×2 ANOVAs on the accuracy data for each group. Both main effects of face age, *F*(1,17) = 10.05, *p*<.01, *η^2^* = .397, and orientation, *F*(1,17) = 11.21, *p*<.005, *η^2^* = .372, were significant for the low-experienced group, whereby children were generally more accurate at discriminating younger adult (*M* = 68.8%) than older adult faces (*M* = 59.6%), irrespective of orientation, and provided more correct responses on upright trials (*M* = 70.5%) than on inverted trials (*M* = 57.9%), irrespective of face age (Figure 4a). Because our primary question concerned experience effects on upright face discrimination, we compared accuracy for younger and older adult faces on upright trials. The comparison was significant, *t*(17) = 2.73, *p*<.05, whereby low-experienced participants were better at discriminating upright younger adult faces (*M* = 74.8%) compared to upright older adult faces (*M* = 66.2%). The ANOVA on the high-experienced group revealed a main effect of orientation, *F*(1,17) = 53.64, *p*<.001, *η^2^* = .759, as well as a Face Age × Orientation interaction, *F*(1,17) = 5.96, *p*<.05, *η^2^* = .260. Unlike low-experienced participants, high-experienced children were more accurate at discriminating upright older adult faces (*M* = 81.6%) compared to upright younger adult faces (*M* = 73.2%), *t*(17) = 2.71, *p*<.05. They also showed a significant inversion effect for both younger adult faces (upright: *M* = 73.2% vs inverted: *M* = 60.8%), *t*(17) = 2.377, *p*<.05, and older adult faces (upright: *M* = 81.6% vs inverted: *M* = 52.3%), *t*(17) = 8.307, *p*<.001, but the size of the inversion cost was larger for older adult faces (*M* = 29.2%) than for younger adult faces (*M* = 12.4%), *t*(17) = 2.44, *p*<.05 (Figure 4b).

To complete the exploration of the differences between the low-experienced and the high-experienced group, we directly compared accuracy data of the two groups for each condition. Between-group comparisons were significant only for the upright older adult faces condition (low-experienced group: *M = *66.2% vs high-experienced group: *M* = 81.6%), *t*(34) = 3.21, *p*<.005, whereas all other comparisons were non-significant (all *p*s >.66). Finally, between-group comparisons of the size of the inversion cost for each face age revealed that, for older adult faces, the inversion cost was larger in the high-experienced group than in the low-experienced group (*M = *29.3% vs 13.9%), *t*(34) = 2.57, *p*<.05, whereas for younger adult faces, the effect was not significantly different in the two groups (*p*>.96).

### Discussion

Results showed that 3-year-old children with limited experience with elderly people were better at differentiating among upright young adult faces than among upright older adult faces. Crucially, this discrimination advantage was not only absent, but was even reversed in children who, from the time of their birth, had extensive contact with elderly adults. These children were more accurate in discrimination of upright older adult faces than upright younger adult faces and, accordingly, they were more experts at processing older adult faces than younger adult faces, as inferred by the size of the inversion effect. These findings add to previous demonstrations that sibling’s experience affects the processing and discrimination of infant and child faces, providing further evidence that experience with faces from a specific age group in the first three years of life can modulate perceptual discrimination abilities for those faces.

**Figure 5:**
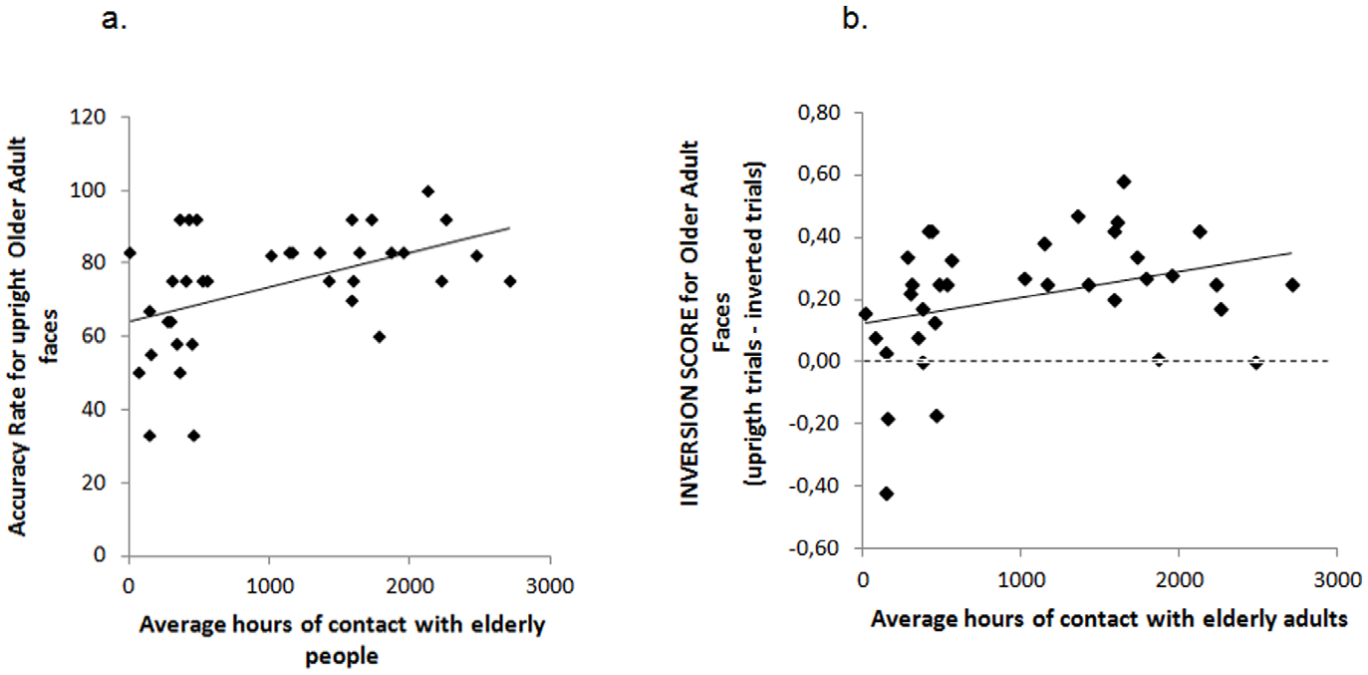
Children’s accuracy rate for upright older adult faces (a) and inversion score for older adult faces (b) collapsed across experience plotted as a function of the average number of hours of contact per year with elderly adults.

An important aspect of the current findings is that children in both the low-experienced and the high-experienced groups showed a significant inversion effect for older adult faces. This implies that even a limited amount of contact with elderly people was sufficient to render children in the low-experienced group as sensitive to the relevant featural/configural cues embedded in older adult faces as they are to the same relevant cues in younger adult faces, and that experience has continuous and cumulative effects on the tuning of the perceptual processes involved in face discrimination and recognition. Accordingly, we found that the average number of hours of contact with elderly people within each year across both groups of children was significantly correlated with the accuracy of upright older adult face discrimination (*r = *.47, *p*<.005) (Figure 5a) and the magnitude of the inversion score for older adult faces (*r = *.33, *p*<.05) (Figure 5b).

### Conclusions

The purpose of the current study was to provide further evidence for the role of social and perceptual experience in modulating age-related face processing biases in adults and young children.

Results showed that adults (Experiment 1) and 3-year-old children (Experiment 2) who had limited contact with elderly adults were better able to distinguish among younger adult faces than among older adult faces. In contrast, discrimination performance of participants who accumulated extensive experience with elderly adults did not differ for the two types of faces. Together, these findings provide further evidence for the existence of a processing advantage for young adult faces over faces of other-age groups in adults and young children, and extend current evidence for a perceptual learning account of such advantage.

Specifically, results extend previous demonstrations of better within-category discrimination for young adult faces over infant and child faces in adults [8], [17], [18] and 3-year-old children [18], [42] by showing that, in the absence of extensive experience, a discrimination advantage for young adult faces is apparent even when these faces are compared with another class of adult faces. Moreover, the comparison between discrimination abilities of adult and child participants in the low-experienced and the high-experienced groups showed that, for both adults and children, visual experience with older adult faces can tune perceptual processing strategies towards this type of faces to the point of eliminating the discrimination advantage for younger adult faces. As for the adults, accuracy measures revealed that, unlike low-experienced participants, high-experienced participants exhibited an inversion effect, which was generalized across both younger and older adult faces, and showed no differences in the discrimination of the two types of face. As for the children, high-experienced participants, unlike the low-experienced, were equally good at discriminating younger and older adult faces. They also manifested an inversion effect for older adult faces, which was larger than that for younger adult faces and larger than the inversion effect for older adult faces in low-experienced participants.

An important difference between the results obtained with adults and children concerns the processing of older adult faces in low-experienced participants. Unlike adults, children in the low-experienced group showed a significant inversion effect for older adult faces, whose size across the whole sample was related to the amount of contact with elderly adults. This suggests that, in children, even a small amount of experience may be sufficient to engender perceptual learning of a specific face age group, and that increasing experience has progressive and cumulative effects on the tuning of the perceptual processes involved in face discrimination and recognition.

Importantly, because both adults and children in the low-experienced groups were selected based on the same criteria for having had limited contact with elderly individuals, the presence of an inversion effect for older adult faces in children but not in adults cannot be explained in terms of differential amount of experience with older adult faces. One possibility is that children’s learning from the limited experience they had with elderly individuals was boosted by motivational mechanisms arising from their tendency to perceive younger and older adults as members of the same broad social group, which may have enhanced children’s interest in older adult faces. Another possibility is that the observed difference in the way low-experienced children and adults processed older adult faces is indicative of a decrease in plasticity of the perceptual processes involved in face recognition between childhood and aduthood, whereby children may have learned more easily than adults from the limited amount of perceptual experience they had with older adult faces. Indeed, earlier studies have reported a loss of plasticity of face processing abilities between childhood and adulthood in response to experience with one face from a specific age group, as in the case of the face of an infant sibling in 3-year-old children or the face of a first-born infant in mothers [17]. The current study would extend this earlier evidence by showing that, although the face processing system remains plastic in response to experience with multiple facial identities from one single age group well into adulthood, such plasticity is limited in comparison to that manifested by young children.

Despite these differences in adults’ and children’s responsivity to the effects of limited experience, there is an interesting similarity between the adults’ and children’s data in the relations between perceptual expertise and discrimination abilities. In children, the presence of an inversion effect for older adult faces did not allow low-experienced participants to overcome their deficit in discriminating among these faces in comparison to younger adult faces. This finding resonates well with the results emerging from the between-groups comparisons of Experiment 1, showing that although high-experienced adults were worse than low-experienced adults at discriminating inverted older adult faces, they were not any better at discriminating these faces in the canonical upright orientation. Together, these findings add to some already existing evidence [18], [21], [55] suggesting that visual experience acquired in adulthood can shape perceptual processes used to discriminate faces without necessarily improving face discrimination abilities, and extend this evidence to children.
